# Prognostic significance of troponin in patients with malignancy (*NIHR Health Informatics Collaborative TROP-MALIGNANCY study*)

**DOI:** 10.1186/s40959-024-00238-w

**Published:** 2024-07-05

**Authors:** Nathan A. Samuel, Alistair Roddick, Ben Glampson, Abdulrahim Mulla, Jim Davies, Dimitri Papadimitriou, Vasileios Panoulas, Erik Mayer, Kerrie Woods, Anoop D. Shah, Sanjay Gautama, Paul Elliott, Harry Hemmingway, Bryan Williams, Folkert W. Asselbergs, Narbeh Melikian, Rajesh Kharbanda, Ajay M. Shah, Divaka Perera, Riyaz S. Patel, Keith M. Channon, Jamil Mayet, Anoop S. V. Shah, Amit Kaura

**Affiliations:** 1 NIHR Imperial Biomedical Research Centre, Imperial College London and Imperial College Healthcare NHS Trust, London, U.K.; 2 NIHR Oxford Biomedical Research Centre, University of Oxford and Oxford University Hospitals NHS Foundation Trust, Oxford, U.K.; 3 National Heart and Lung Institute, Imperial College London, London, U.K.; 4 Department of Surgery & Cancer, Imperial College London, London, UK; 5 Hospitals Biomedical Research Centre, NIHR University College London, University College London and University College London Hospitals NHS Foundation Trust, London, U.K.; 6 Health Data Research UK, London Substantive Site, London, U.K.; 7 NIHR King’s Biomedical Research Centre, King’s College London and King’s College Hospital NHS Foundation Trust, London, U.K.; 8 NIHR King’s Biomedical Research Centre, King’s College London and Guy’s and St Thomas’ NHS Foundation Trust, London, U.K.; 9 London School of Hygiene Tropical Medicine, London, U.K.; 10 Imperial College Healthcare NHS Trust, Hammersmith Hospital, NHLI offices, B Block 2nd Floor, Du Cane Road, W12 0HS London, U.K.

**Keywords:** Troponin, Malignancy, Cancer, Cardio-oncology, Mortality, Biomarkers

## Abstract

**Background:**

Cardiac troponin is commonly raised in patients presenting with malignancy. The prognostic significance of raised troponin in these patients is unclear.

**Objectives:**

We sought to investigate the relation between troponin and mortality in a large, well characterised cohort of patients with a routinely measured troponin and a primary diagnosis of malignancy.

**Methods:**

We used the National Institute for Health Research (NIHR) Health Informatics Collaborative data of 5571 patients, who had troponin levels measured at 5 UK cardiac centres between 2010 and 2017 and had a primary diagnosis of malignancy. Patients were classified into solid tumour or haematological malignancy subgroups. Peak troponin levels were standardised as a multiple of each laboratory’s 99th -percentile upper limit of normal (xULN).

**Results:**

4649 patients were diagnosed with solid tumours and 922 patients with haematological malignancies. Raised troponin was an independent predictor of mortality in all patients (Troponin > 10 vs. <1 adjusted HR 2.01, 95% CI 1.73 to 2.34), in solid tumours (HR 1.84, 95% CI 1.55 to 2.19), and in haematological malignancy (HR 2.72, 95% CI 1.99 to 3.72). There was a significant trend in increasing mortality risk across troponin categories in all three subgroups (*p* < 0.001).

**Conclusion:**

Raised troponin level is associated with increased mortality in patients with a primary diagnosis of malignancy regardless of cancer subtype. Mortality risk is stable for patients with a troponin level below the ULN but increases as troponin level increases above the ULN in the absence of acute coronary syndrome.

**Supplementary Information:**

The online version contains supplementary material available at 10.1186/s40959-024-00238-w.

## Introduction

The last few decades have seen the emergence of increasingly potent pharmacological and non-pharmacological cancer therapies which improve survival but also have significant cardiovascular toxicity with short and long-term cardiac sequalae [1]. The mechanisms underlying the associations between cancer and cardiovascular disease are multifactorial and include direct effects of cancer on the myocardium, the subsequent effect of potent cancer therapy resulting in cardiac injury, and the shared risk factors associated with both cardiovascular disease and cancer such as obesity and smoking [2]. There is also emerging evidence of the deleterious impact of cardiovascular disease on cancer prognosis [3, 4]. 

Biochemical evidence of myocardial injury, defined as an elevation in cardiac troponin concentration, is widely used in the diagnosis of myocardial infarction and risk stratification in patients with suspected acute coronary syndrome [5, 6]. Cardiac troponin levels are also higher in patients with other cardiac disease and influenced significantly by co-existing multimorbidity [7]. Elevated cardiac troponins and adverse cardiac remodelling have also been associated with patients receiving cardiotoxic chemotherapy [8–10]. Smaller studies have also shown lower survival in cancer patients with elevated cardiac troponin concentrations [11]. 

In this large retrospective cohort study, we evaluate the association between cardiac troponin concentrations and survival. We further explore the interaction between troponin concentrations and survival by cancer type.

## Methods

### Data sources

The National Institute for Health Research (NIHR) Health Informatics Collaborative project was established to facilitate the re-use of routinely collected clinical data for hypothesis driven clinical research [12–15]. Five hospital trusts (Imperial College Healthcare, University College Hospital, Oxford University Hospital, King’s College Hospital and Guys and St Thomas’ Hospital) contributed data to the project. All five hospitals are tertiary centres with accident and emergency departments. These centres provided a broad set of data including patient demographics, blood results, procedural data, and mortality. Between 2010 (2008 for University College Hospital) and 2017, we enrolled 257,948 consecutive patients who had a troponin measured. In cases where patients had multiple hospital episodes of care with troponin measurements, only the first episode was eligible. 134,517 (52.1%) patients had a hospital admission and therefore had International Statistical Classification of Diseases and Related Health Problems (ICD) discharge codes [16]. The study was registered at ClinicalTrials.gov, NCT03507309.

### Population

**Fig. 1:**
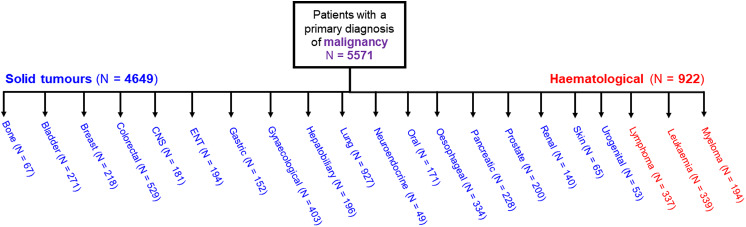
Flowchart of patients included in the study

We included 5571 patients who had a malignancy-related hospitalisation and underwent at least one troponin test during their hospital stay. Malignancy-related hospitalizations were identified by a primary ICD-10 code between C00 and C96. Patients with malignancy were then classified into those with solid tumours (*N* = 4649) and those with haematological malignancy (*N* = 922) (Fig. 1).

### Troponin definitions

Details of troponin assays used at participating cardiac centres are included in Supplementary Table 1. These troponin assay tests yielded results in different measurable ranges, with unique cut-off points for the 99th centile of the upper limit of normal (ULN), which are measured in ng/L. Due to differences in the ULN between troponin assays, we standardised the results by using the ratio of the observed troponin value divided by the ULN for each troponin assay. Since the outcome is expressed as a ratio, the troponin values are devoid of units. For example, a troponin concentration 10-fold higher than the assay specific ULN was assigned a value of 10. For patients who only had one troponin measured, the peak troponin was based on this measurement. For patients who had multiple troponin measurements in the same hospital episode of care, the peak troponin value was the largest troponin value of all the measurements. For patients with multiple episodes of care during which a troponin was measured, only the first episode of care was used for analyses.

### Outcomes and follow-up

The primary outcome was 1-year all-cause mortality. Patients were followed up until death or censoring in April 2017. Life status was determined using routinely collected data linked to the Office of National Statistics and to the national registry of deaths.

### Statistical analysis

Categorical results are presented as number and percentages, continuous data as median with interquartile ranges (IQR).

Survival analyses were conducted using standardised troponin level both categorised into three groups (< 1, 1–10, and > 10), and as a continuous variable. Kaplan-Meier cumulative mortality plots were constructed to show the results and the log-rank test was used to compare survival according to troponin level. Univariable and multivariable cox proportional hazards analyses were used to evaluate the effect of independent variables on mortality. The proportionality of hazards assumption was evaluated with the use of log-log survival curves and by assessing the correlation between scaled Schoenfeld residuals and length of follow-up. Troponin as a continuous variable was analysed through modelling non-linear relationships using restricted cubic splines with three knots. A P value of less than 0.05 (two-tailed) was considered statistically significant. Statistical analyses were performed using SPSS 25 (SPSS Inc., Chicago, Illinois) and R Statistical Software version 3.4.3 (R Development Core Team, California, USA).

## Results

The flow of patients through the study is shown in Fig. 1. 5571 patients had a primary diagnosis of malignancy and comprised of twenty-one different cancer types. 4649 patients were diagnosed with solid tumours and 922 patients were diagnosed with haematological malignancies.

### Baseline characteristics

Baseline characteristics of patients by troponin level are shown in Table 1. Patients with raised troponin had a higher burden of cardiovascular comorbidities compared to patients with a troponin level below the ULN. Patients with a raised troponin were also more likely to be diagnosed with ACS and undergo angiography, although this only applied to a small number of patients in our cohort (~ 2%). Baseline characteristics of patients by cancer subgroup are shown in Table 2. Patients with solid tumours were older, but had a similar burden of cardiovascular comorbidities when compared to patients with haematological malignancy.

**Table 1:** Baseline characteristics of patients by troponin category. Troponin values are given as multiples of upper limit of normal (ULN)

Variable	Troponin < 1	Troponin 1–10	Troponin > 10
	*N* = 3000	*N* = 2082	*N* = 489
Age (years)	70 (61 to 78)	70 (61 to 78)	62 (47 to 71)
Male sex n(%)	1617 (54)	1236 (59)	265 (54)
C-reactive protein (mg/L)	40 (9 to 112)	47 (11 to 120)	41 (9 to 128)
Creatinine (µmol/L)	72 ( 59 to 94)	75 (59 to 101)	75 (60 to 100)
Haemoglobin (g/dL)	11.3 (9.9 to 12.9)	10.9 (9.5 to 12.6)	9.6 (8.3 to 11.0)
Platelet count (10^9^/L)	245 (187 to 319)	231 (166 to 315)	67 (26 to 170)
White cell count (10^9^/L)	9.8 (7.2 to 13.3)	9.5 (6.6 to 13.1)	5.0 (1.3 to 12.6)
Sodium (mmol/L)	138 (135 to 140)	138 (135 to 140)	138 (135 to 141)
Potassium (mmol/L)	4.3 (3.9 to 4.6)	4.2 (3.8 to 4.6)	4.0 (3.6 to 4.4)
Atrial fibrillation n(%)	199 (7)	339 (16)	77 (16)
Heart failure n(%)	74 (2)	160 (8)	22 (4)
Previous MI n(%)	234 (8)	265 (13)	69 (14)
Acute coronary syndrome n(%)	11 (0.4)	32 (1.5)	79 (16.2)
Angiogram n(%)	25 (0.8)	18 (0.9)	33 (6.7)
Acute revascularisation n(%)	4 (0.1)	5 (0.2)	19 (3.9)
Diabetes mellitus n(%)	374 (13)	356 (17)	79 (16)
Hypertension n(%)	625 (21)	686 (33)	148 (30)
Chronic kidney disease > stage 2 n(%)	68 (2)	136 (7)	39 (8)
Chronic obstructive pulmonary disease n(%)	157 (5)	185 (9)	42 (9)

Categorical results are presented as number (percentage), continuous data as median (interquartile range)

**Table 2:** Baseline characteristics of patients by malignancy type. Troponin values are given as multiples of upper limit of normal (ULN)

Variable	Malignancy type	
	Solid tumours	Haematological
	*N* = 4649	*N* = 922
Age (years)	71 (62 to 78)	64 (51 to 73)
Male sex n(%)	2581 (56)	537 (58)
C-reactive protein (mg/L)	48 (11 to 123)	36 (8 to 111)
Creatinine (µmol/L)	73 (59 to 95)	78 (62 to 112)
Haemoglobin (g/dL)	11.2 (9.8 to 12.8)	9.7 (8.5 to 11.2)
Platelet count (10^9^/L)	246 (186 to 326)	96 (31 to 204)
White cell count (10^9^/L)	10.0 (7.4 to 13.5)	5.4 (1.6 to 10.3)
Sodium (mmol/L)	138 (135 to 140)	138 (135 to 141)
Potassium (mmol/L)	4.3 (3.9 to 4.6)	4.0 (3.6 to 4.4)
Atrial fibrillation n(%)	521 (11)	94 (10)
Heart failure n(%)	202 (4)	54 (6)
Previous MI n(%)	482 (10)	86 (9)
Acute coronary syndrome n(%)	100 (2)	22 (2)
Angiogram n(%)	65 (1.4)	11 (1.2)
Acute revascularisation n(%)	24 (0.5)	4 (0.4)
Diabetes mellitus n(%)	677 (15)	132 (14)
Hypertension n(%)	1250 (27)	209 (23)
Chronic kidney disease > stage 2 n(%)	213 (5)	30 (3)
Chronic obstructive pulmonary disease n(%)	320 (7)	64 (7)

Categorical results are presented as number (percentage), continuous data as median (interquartile range)

### Relation between troponin and mortality in all patients with malignancy

The median follow-up in the cohort was 14 months (IQR 2–39 months). At 1-year follow-up, 2361 (42%) of patients died.

**Fig. 2:**
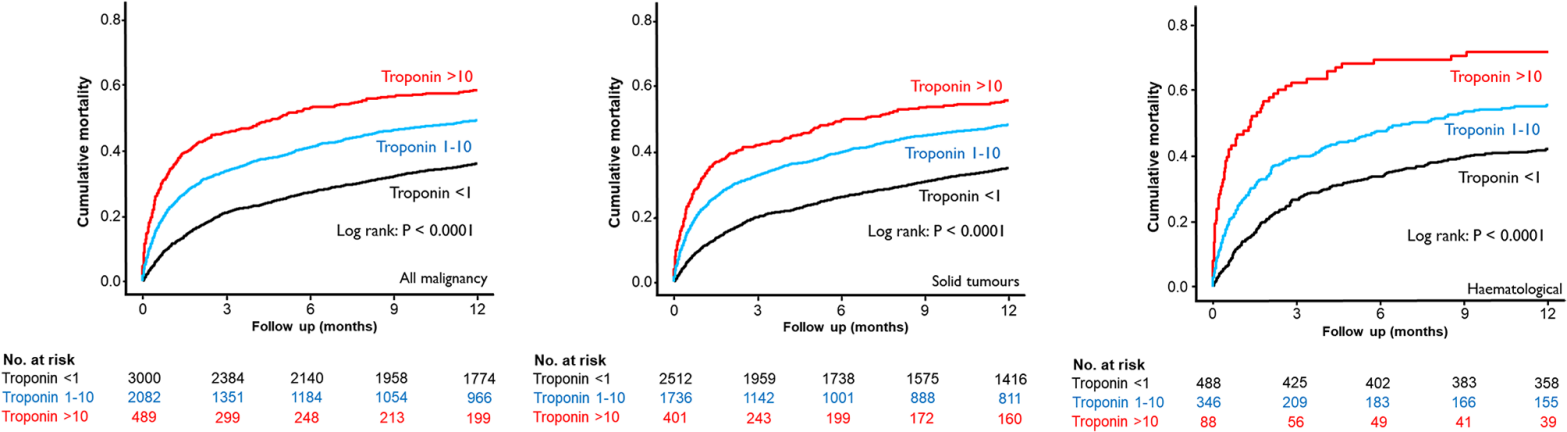
Kaplan-Meier cumulative mortality plots for all-cause mortality. Troponin values are given as multiples of upper limit of normal (ULN)

Figure 2 shows Kaplan-Meier cumulative mortality plots for patients stratified by troponin category (xULN). Patients with a troponin level > 1 had reduced survival compared to patients with a troponin level 1–10, patients with a troponin > 10 had the worst mortality (Fig. 2A). In patients with solid tumours and haematological malignancy, each troponin category was associated with an increasing mortality (Fig. 2B and C).

**Fig. 3:**
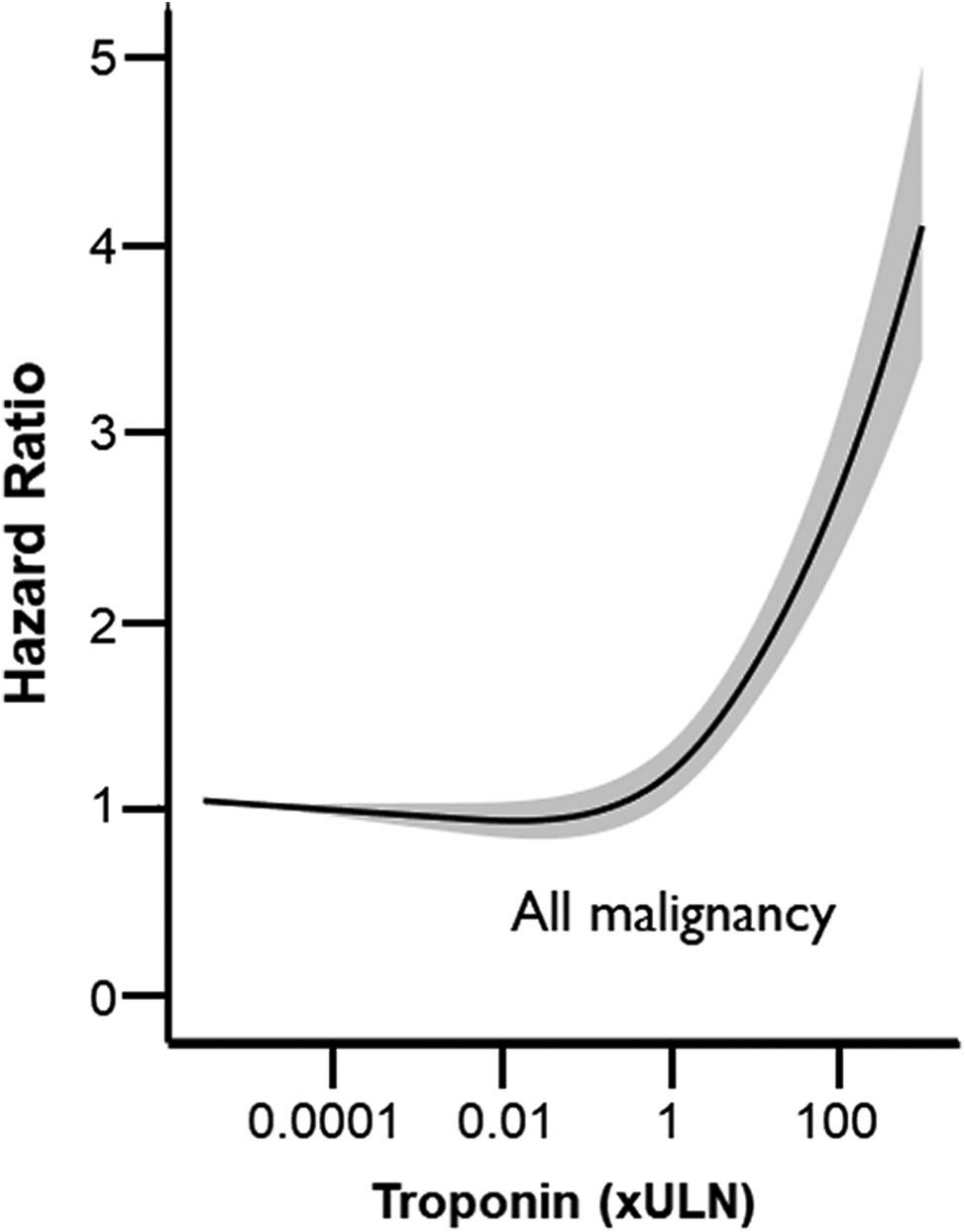
Relation between troponin level and mortality risk across all malignancies. Adjusted restricted cubic splines for the association between standardised peak troponin and 1-year all-cause mortality. Adjusted for age, sex, c-reactive protein, haemoglobin, platelet count, white cell count, acute coronary syndrome, diabetes mellitus, heart failure, chronic kidney disease, chronic obstructive pulmonary disease, and atrial fibrillation

In univariable cox regression analysis, patients with a raised troponin (troponin 1–10 or > 10) had a significantly higher all-cause mortality compared to patients with a negative troponin (< 1) (troponin 1–10 vs. <1, HR 1.60, 95% CI 1.46 to 1.74, and troponin > 10 vs. <1, HR 2.22, 95% CI 1.95 to 2.54, p-value for overall association < 0.001) (Supplementary Table 2). After adjustment for patient demographics, clinical factors, and comorbidities; raised troponin was an independent predictor of mortality (troponin 1–10 vs. <1, HR 1.56, 95% CI 1.42 to 1.71, troponin > 10 vs. < 1, HR 2.01, 95% CI 1.73 to 2.34, p-value for trend < 0.001). Figure 3 shows the relation between standardised peak troponin level and hazard ratio among all patients with malignancy. The hazard ratio for all-cause mortality was similar among patients with a peak troponin below the ULN. Above the ULN, higher troponin multiples were associated with increasing mortality risk. Mortality risk increases as troponin level increases before and after adjustment for patient demographics, clinical factors, and comorbidities. This relationship between troponin level and mortality was present across both troponin I and troponin T assays (Supplementary Fig. 3), as well as both standard sensitivity and high-sensitivity troponin assays (Supplementary Fig. 4).

### Relation between troponin and mortality in different types of malignancy

**Fig. 4:**
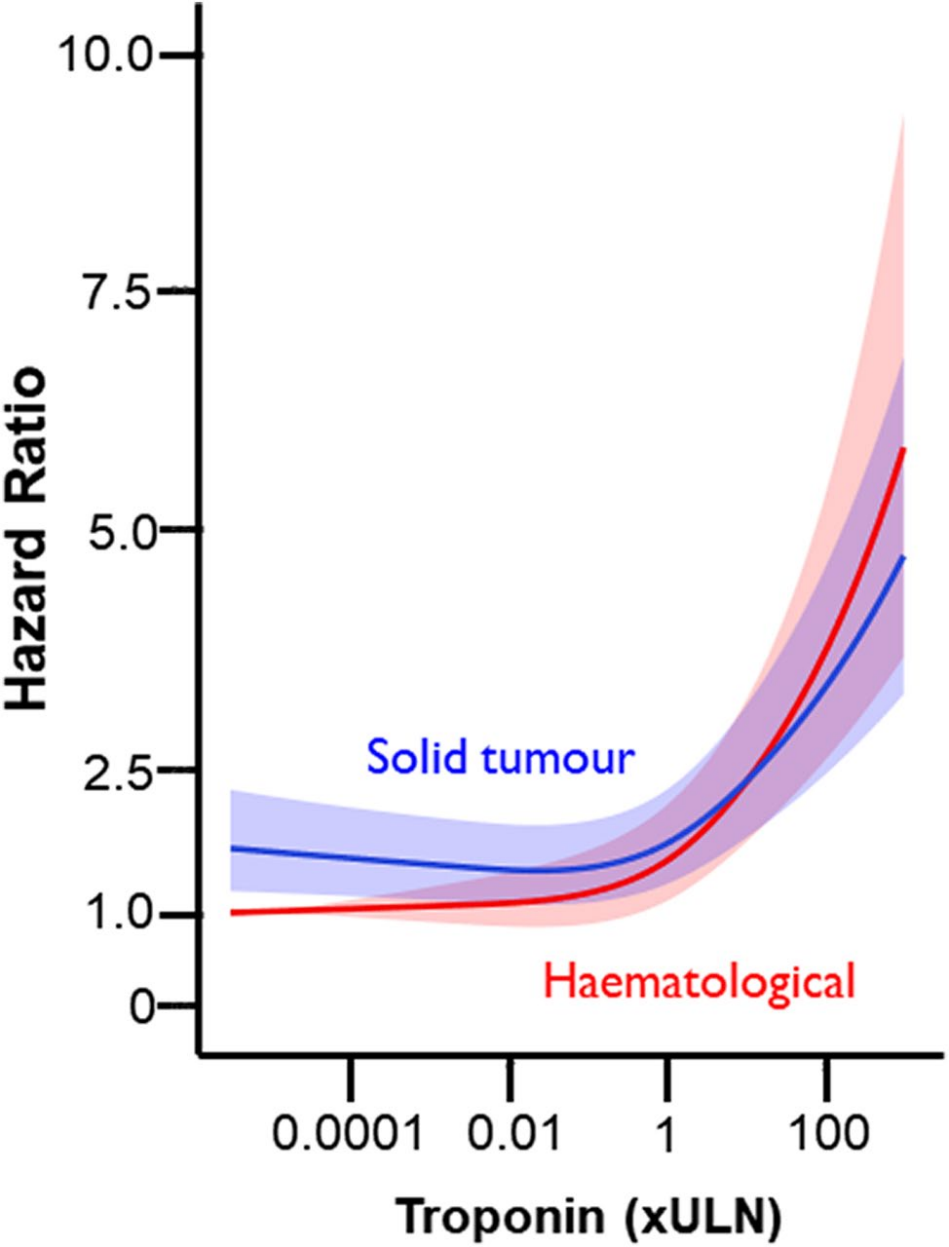
Relation between troponin level and mortality risk in patients by cancer type. Adjusted restricted cubic splines for the association between standardised peak troponin and 1-year all-cause mortality. Adjusted for age, sex, c-reactive protein, haemoglobin, platelet count, white cell count, acute coronary syndrome, diabetes mellitus, heart failure, chronic kidney disease, chronic obstructive pulmonary disease, and atrial fibrillation

Figure 4 shows the relation between troponin level and hazard ratio among patients with malignancy by cancer type. At lower troponin levels, mortality risk was higher among patients with solid tumours compared to patients with haematological malignancy for a given troponin level. At higher troponin levels, mortality risk was higher among patients with haematological malignancy compared to patients with solid tumours for a given troponin level (Supplementary Table 2).

**Fig. 5:**
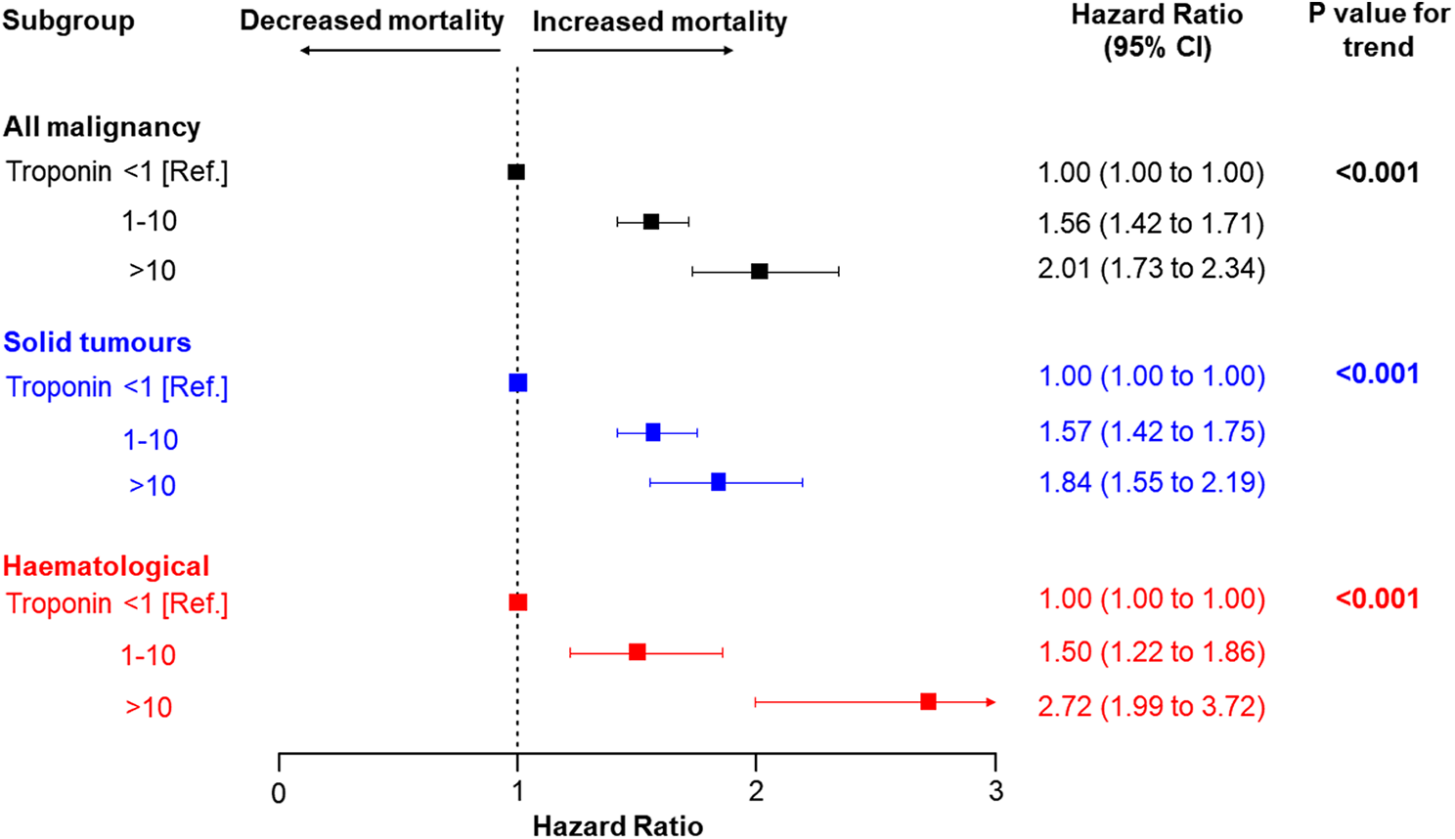
Adjusted hazard ratios associated with raised troponin categories (above the ULN) compared to troponin below the ULN in difference cancer types. Point estimates and 95% confidence intervals for adjusted 1-year hazard ratios are visually represented. Patients with a raised troponin category (either 1–10 or > 10) were compared to patients with a troponin level below the ULN (< 1). In all three analyses, results were adjusted for age, sex, c-reactive protein, haemoglobin, platelet count, white cell count, acute coronary syndrome, diabetes mellitus, heart failure, chronic kidney disease, chronic obstructive pulmonary disease, and atrial fibrillation

Figure 5 shows hazard ratios associated with a raised troponin compared to a troponin level below the ULN. In all cancer subgroups, a raised troponin was associated with increased mortality. Restricted cubic splines showing the relation between troponin level and mortality by troponin type and assay sensitivity are included in the supplementary material.

## Discussion

We have shown that troponin is an independent predictor of mortality in both haematological and solid tumour malignancy. Another key finding of our study is that mortality risk increases as troponin level increases above the ULN.

The strength of this study is that it relates to a large, well characterised, unselected cohort of patients with a troponin measurement in the clinical setting using routinely collected electronic health record data. The large size of the study population allowed adjustment of the cox regression analyses for many clinically relevant variables without being event limited.

The results support the findings of Hollebecque et al. which showed that raised troponin was associated with reduced survival in 463 patients enrolled in phase 1 trials, followed for twenty-four months [17]. These results also support the findings from Pavo et al. which showed hsTnT was associated with increased mortality (HR 1.21, 95% CI 1.13 to 1.32, *P* < 0.001) after adjustment for age, tumour stage, tumour entity, cardiac status, and GFR in 555 patients who were not receiving cardiotoxic chemotherapy, followed for twenty-five months [18]. The present study extends and strengthens these findings by illustrating the relation between troponin and increased mortality in a multi-centre study with much larger numbers, enabling adjustment for a very wide range of comorbidities and ACS treatment. When assessing the relationship between troponin level and mortality, validating findings across different populations is indeed crucial to ensure that conclusions drawn from one group can be generalized to others. When it comes to using troponin levels to predict mortality in patients with malignancy, it is essential to consider whether this prediction is merely an extension of findings observed in the general population [19], or if it holds true specifically for cancer patients. We therefore included a relatively large sample of cancer patients, with diverse types of cancer, to ensure our findings were representative. Moreover, we employed statistical techniques to assess whether the predictive power of troponin levels holds true in cancer patients independently of other factors. This included adjusting for confounding variables such as age, gender, and comorbidities, to isolate the impact of troponin levels on mortality specifically in cancer patients. We also performed subgroup analyses within the cancer population to examine whether the predictive ability of troponin levels varies across different cancer types. As troponin levels predicted mortality across subgroups within the cancer population, this provides stronger evidence that this association is not merely reflective of data from the general population.

### The prognostic significance of troponin in different types of malignancy

Although we found subtle differences in the prognostic significance of raised troponin among solid tumour and haematological malignancy subgroups, the overall relation between increasing troponin level and increased mortality persisted in both subgroups. It is possible that patients with a raised troponin represent a population with undiagnosed ACS. ACS is more likely to be underdiagnosed in patients with malignancy, especially in advanced stages, since they often have comorbidities that predispose them to complications arising from dual antiplatelet therapy or invasive procedures such as angiography or percutaneous coronary intervention. Thrombocytopenia, prior mediastinal radiation, or hypercoagulable states can each individually or collectively affect the ability to administer dual anti-platelets or perform coronary angiography safely [20]. Thrombocytopenia increases the risk of bleeding complications during invasive procedures such as angiography, especially whilst also on dual antiplatelet therapy. Prior mediastinal radiation therapy can cause tissue fibrosis and vascular damage, making catheterisation technically challenging and increasing the risk of complications such as perforation. Furthermore, radiation-induced vascular damage, including endothelial dysfunction, may predispose patients to thrombosis. This risk is further compounded if the patient is also receiving dual antiplatelet therapy. Hypercoagulable states can also predispose patients to thrombotic events.

Troponin levels could also signify non-coronary cardiac pathology including heart failure in the context of malignancy and its therapy-associated side effects [8, 21]. Another possibility is that within each subgroup, troponin could reflect cancer prognosis in some patients [2] while it may identify other patients with pre-existing subclinical CVD which has been destabilised by cancer-induced inflammation, neurohormonal activation or cancer therapy-associated side effects [22]. A combined explanation is that regardless of cancer type, a final common pathway exists related to advanced disease, frailty, comorbidities, and diminished organ level (including cardiac) reserve that is identified by a raised troponin, which is associated with adverse prognosis.

### Clinical implications

Measuring troponin in patients with malignancy can aid clinicians in risk prediction. The European Society of Cardiology recommends that troponin measurement may be considered in addition to clinical history taking and examination for the purposes of risk stratification before starting cardiotoxic cancer therapy and to monitor cardiac function during treatment [23]. There have been no studies investigating the utility of a cardiac biomarker-guided approach to management in patients with malignancy who are not receiving cardiotoxic cancer therapy. The advantages to such an approach are the wide availability, accuracy, reproducibility, and high sensitivity of cardiac biomarkers such as troponin. These data suggest that troponin may be more widely useful in the risk stratification of patients with cancer. Although the appropriate management of patients in response to raised troponin in the absence of ACS is not clear, stratification of clinical risk of mortality can be helpful in general decision making.

### Study limitations

The main limitation of our study is confounding by indication. All patients included in our study had a troponin level measured for clinical reasons, therefore, patients in our cohort may represent those at higher cardiovascular risk than the general population of patients with malignancy. We used discharge codes for diagnosing malignancy and cancer types, but these final diagnoses were not independently verified. We did not have data on medications; therefore, we were unable to investigate the impact of patients receiving cardiotoxic cancer therapy on the results and we also couldn’t determine the proportion of patients who were receiving preventive therapy for CVD. We did not have data on mode of death; therefore, we were unable to study the endpoints of cardiovascular-related death and cancer-related death.

## Conclusion

Raised troponin level is associated with increased mortality in patients with malignancy regardless of cancer subtype. Mortality risk is stable for patients with a troponin level below the ULN but increases as troponin level increases above the ULN. Measuring troponin in patients with solid tumours and haematological malignancy can aid clinicians in risk prediction, since a high troponin level is cause for concern in this population even in the absence of ACS.

**Figure 1. Flowchart of patients included in the study**.

### Electronic supplementary material

Below is the link to the electronic supplementary material.

Supplementary Material 1

Supplementary Material 2

Supplementary Material 3

## Data Availability

Supplementary data is provided in the submission; the raw data is not currently available for public use.
