# Supplementary material for: Prognostic significance of troponin in patients with malignancy (NIHR Health Informatics Collaborative TROP-MALIGNANCY study)
Source: Cardiooncology. 2024 Jul 5;10:41. doi: 10.1186/s40959-024-00238-w (PMC11225146; doi:10.1186/s40959-024-00238-w)
Supplement: Supplementary file 2 — Supplementary Material 2 [file 40959_2024_238_MOESM2_ESM.docx]

| **Assay Manufacturer / Platform** | **Gender-specific** | **99^th^ percentile of the ULN (ng/L)** | **Troponin** | **No. of patients** |
| --- | --- | --- | --- | --- |
| **High-sensitivity troponin assays** |  |  |  |  |
| Roche Cobas | No | 14  (results reported with ULN of 10) | Tn T | 1107 |
| Roche Elecsys | No | 14  (results reported with ULN of 13) | Tn T | 1251 |
| Abbott i-STAT | Female | 15 | Tn I | 250 |
| Abbott i-STAT | Male | 34 | Tn I | 330 |
| Abbott Architect | No | 40 | Tn I | 1100 |
| **Standard sensitivity troponin assays** | |  |  |  |
| Roche Elecsys cTnT | No | 30 | Tn T | 299 |
| Abbott cTnI | No | 32 | Tn I | 1234 |

**Supplementary tables**

**Supplementary Table 1.** **Troponin assays at participating cardiac centres.**

| **Subgroup** | **Univariable Analysis** | | | | **Multivariable Analysis** | | | |
| --- | --- | --- | --- | --- | --- | --- | --- | --- |
|  | **HR** | **95% CI** | **X^2*^** | **P value*** | **HR** | **95% CI** | **X^2**^** | **P value**** |
| **All patients** |  |  |  |  |  |  |  |  |
| Troponin <1 [Ref.] | 1.00 | 1.00 to 1.00 | 187.8 | **<0.001** | 1.00 | 1.00 to 1.00 | 120.8 | **<0.001** |
| 1-10 | 1.60 | 1.46 to 1.74 |  |  | 1.56 | 1.42 to 1.71 |  |  |
| >10 | 2.22 | 1.95 to 2.54 |  |  | 2.01 | 1.73 to 2.34 |  |  |
|  |  |  |  |  |  |  |  |  |
| **Solid tumour** |  |  |  |  |  |  |  |  |
| Troponin <1 [Ref.] | 1.00 | 1.00 to 1.00 | 142.3 | **<0.001** | 1.00 | 1.00 to 1.00 | 83.8 | **<0.001** |
| 1-10 | 1.61 | 1.46 to 1.77 |  |  | 1.57 | 1.42 to 1.75 |  |  |
| >10 | 2.10 | 1.81 to 2.44 |  |  | 1.84 | 1.55 to 2.19 |  |  |
|  |  |  |  |  |  |  |  |  |
| **Haematological** |  |  |  |  |  |  |  |  |
| Troponin <1 [Ref.] | 1.00 | 1.00 to 1.00 | 50.4 | **<0.001** | 1.00 | 1.00 to 1.00 | 37.7 | **<0.001** |
| 1-10 | 1.55 | 1.27 to 1.89 |  |  | 1.50 | 1.22 to 1.86 |  |  |
| >10 | 2.83 | 2.13 to 3.75 |  |  | 2.72 | 1.99 to 3.72 |  |  |

**Supplementary Table 2.** **Univariable and multivariable analysis for 1-year all-cause mortality in patients with malignancy**

*X^2^ and P-value corresponds to Likelihood ratio test for global association

**X^2^ and P-value corresponds to Likelihood ratio test for trend

**Supplementary figures**


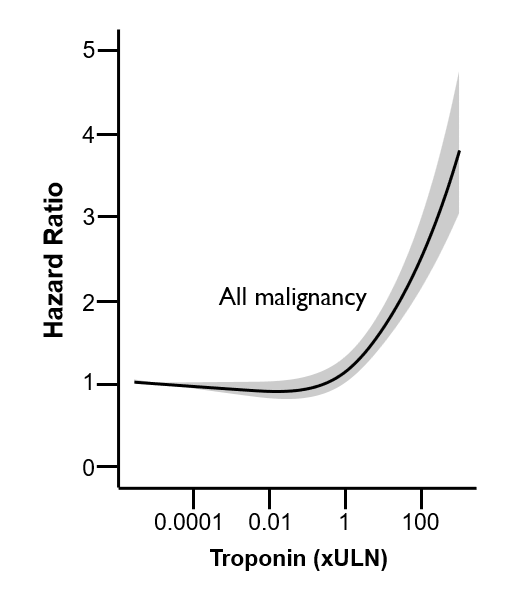


**Supplementary Figure 1. Unadjusted restricted cubic splines for the association between standardised peak troponin and all-cause mortality**


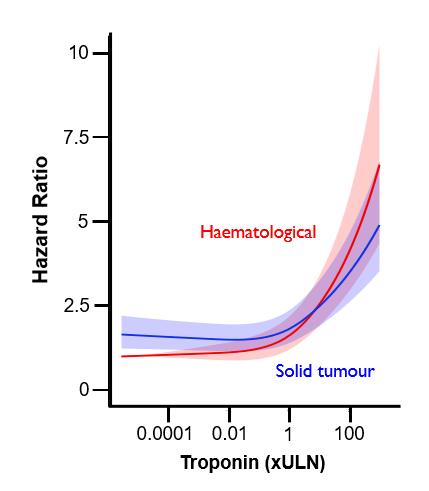


**Supplementary Figure 2. Unadjusted restricted cubic splines for the association between standardised peak troponin and all-cause mortality**

**
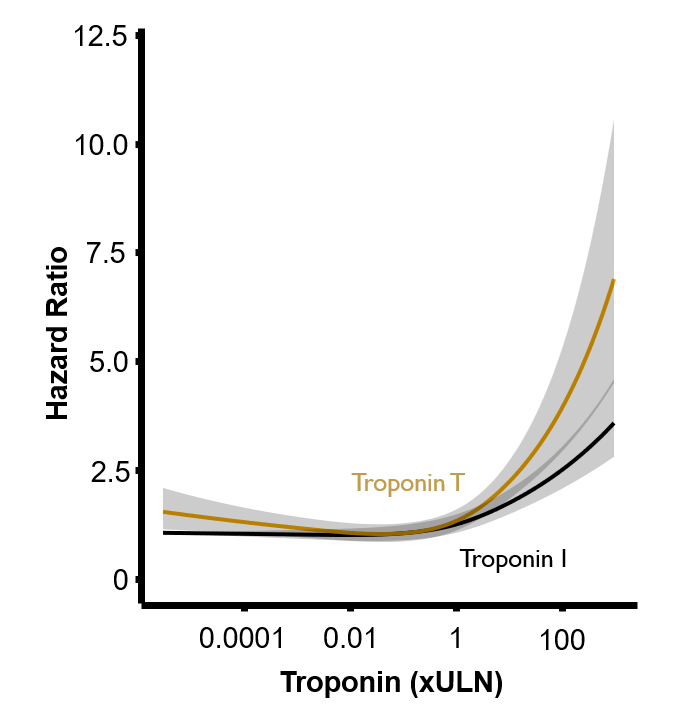
**

**Supplementary Figure 3.** **Adjusted restricted cubic splines for the association between standardised peak troponin and all-cause mortality by troponin type**


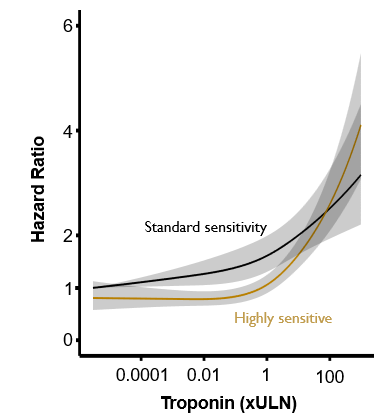


**Supplementary Figure 4.** **Adjusted restricted cubic splines for the association between standardised peak troponin and all-cause mortality by assay sensitivity**
