# Supplementary material for: Prognostic significance of troponin in patients with malignancy (NIHR Health Informatics Collaborative TROP-MALIGNANCY study)
Source: Cardiooncology. 2024 Jul 5;10:41. doi: 10.1186/s40959-024-00238-w (PMC11225146; doi:10.1186/s40959-024-00238-w)
Supplement: Supplementary file 3 — Supplementary Material 3 [file 40959_2024_238_MOESM3_ESM.docx]

**NIHR Health Informatics Collaborative TROP-MALIGANCY Study**

**DATA COLLECTION PLAN**

# DATABASE PROTOCOL

## Introduction

This document outlines the specifications and procedures for the NIHR Health Informatics Collaborative (NIHR HIC) Cardiovascular research database and defines the processes for the collection of the NIHR HIC Cardiovascular research data and onward sharing with researchers. The central research database is held within Imperial College Healthcare NHS Trust (ICHNT).

Data for all patients receiving a troponin test are collected locally at the following Trusts and submitted pseudonymously to ICHNT:

- Imperial College Healthcare NHS Trust
- University College London Hospitals NHS Foundation Trust
- Oxford University Hospitals NHS Foundation Trust
- Kings College Hospital NHS Foundation Trust
- Guys and St Thomas’ Hospital NHS Foundation Trust

This document covers the processes for local collection of data at all sites. The main database and systems are hosted at ICHNT as the lead organisation for the Cardiovascular Theme.

## NIHR HIC Cardiovascular Database Definitions

#### Local data store

Each Trust will have a local store of NIHR HIC Cardiovascular data; this collects their information in an identifiable form from clinical systems. The data will then be de-identified within the local NHS Trust. These de-identified data will be passed to the central research database.

#### Research database

The research database will contain only de-identified information. This database combines the data from each site (including ICHNT). The database will contain some secondary patient identifiers (e.g. date of procedure, date of death) which will not be made directly available to researchers. A further anonymised view of the data will be prepared by ICHNT staff which will involve converting all dates to the number days since the first troponin test. This view of the data will contain only the variables necessary to complete the study rather than the full database.

## Procedures for local data collection into local stores

Data will be collected automatically from primary clinical systems within each Trust. NHS staff will enter data into clinical systems during routine clinical care of patients, or data will be generated as a result of clinical tests. Data in the primary clinical systems will be processed in accordance with each Trusts clinical guidelines and subject to local quality and governance procedures. Data will be extracted automatically and validated processes for data extraction, transformation and loading (ETL) have been designed to import the data into the local secure data stores.

### Access to local data stores

Access to identified data will be limited to those justified and approved by the local information governance teams and will always be NHS staff in accordance with the duty of confidentiality required by law. Anonymisation procedures will be automated after implementation.

Local data stores will be the only areas that hold any patient identifiers. De-identification is completed at this stage prior to passing data into any research databases and datasets.

### De-identification

The data will be pseudonymised locally within each Trust; anonymisation processes will be automated and set up by NHS staff in accordance with:

- advice from local information governance procedures
- the HIC Standard Operating Procedure (SOP) for data sharing and anonymisation
- the Clinical Data transfer policy

The data items summarised in Table 1 will be anonymised. Anonymisation will be approved locally by information governance teams before data are sent externally to ICHNT. De-identified data are then shared in accordance with the overarching data sharing agreement.

| **Demographic** | **Anonymisation** |
| --- | --- |
| Local Identifier | Provided if NHS number is missing |
| NHS Number | Use local pseudonymisation algorithm (key to be retained at source)  Rename field to subject ID |
| Family Name | To be removed – LOCAL use |
| Given Name | To be removed – LOCAL use |
| Date of Birth | YYYY |
| Date of Death | DD-MM-YYYY |

**Table 1. Anonymisation of data elements**

Each site will hold two versions of the database, one identifiable and one with de-identified, pseudonymised data. The de-identified version is for use in research and shared with the central research database at ICHNT. The identifiable database is held so that if necessary patients can be re-identified if it is of importance to re-contact the patient via their care team.

NHS numbers and hospital numbers are pseudonymised using locally approved procedures. Names are removed from the dataset and date of birth is transformed to year of birth. Date of death is shared, however, is converted in to relevant survival rates on provision of data to researchers. Researchers will never see the full date of death or be able to calculate it from other information (all dates are provided as delta for first troponin). The provision of date of death has been agreed by each of the information governance offices at each of the sites in the following de-identification and anonymisation protocol for the study:

- The exact date of death is required to evaluate mortality after diagnosis.
- Patients presenting with suspected acute coronary syndromes are likely to have a high frequency of cardiac events and a high short-term mortality rate. An accurate measure of death is therefore required to fully evaluate this.
- To redact the date of death to year only would misrepresent the survival of these patients, particularly for those who survive for less than one year. The clinical leads at our BRCs have underlined the importance of having the date in full for the study.

### Data validity and quality

Prior to pseudonymisation within the clinical systems, NHS number, date of birth and patient names will be automatically checked to remove duplication of patients.

Samples of data will be clinically validated by members of the clinical team to ensure that the transformation process is correct and data are attributed to the correct patients prior to pseudonymisation. After clinical validation is complete, the data will be transformed to a standardised XML format and validated (Figure 1).


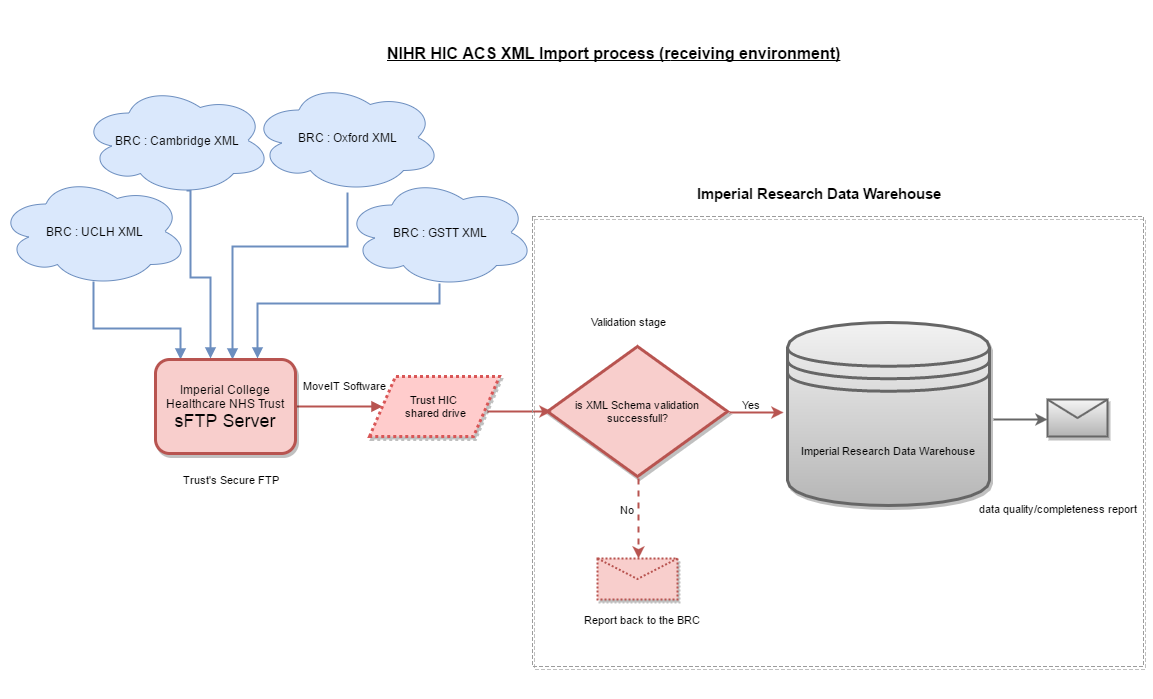


**Figure 1.** Data import and validation process.

### Data sharing between Trusts

Data are shared in accordance with the SOP for data sharing. Data are encrypted in transit via sFTP on the N3 network (Figure 1). The sFTP is set up and hosted by ICHNT.

## Procedures for secure research database

### Data validity and quality

ICHNT validate the data to ensure that the structure, data items, units and data types are in accordance with the standardised data model. Data will be rejected if validation fails. On rejection, the files will be archived and the data manager will contact the data provider to review the submission and resend once corrected.

Once imported into the secure research database, data are subject to clinical validation by Cardiovascular clinical experts; these validations will be completed by the clinical researchers using de-identified data. Data will be reviewed for data completeness, spread and actual data point values. If data appears invalid it will be rejected.

Data quality reports will be generated and provided to the research team and local data provider after each submission. These will be reviewed after each submission to ensure all areas are populated.

### Research Database software

The database will be built using Microsoft SQL server 2014, Microsoft’s principle database management system software. The installation of the software was carried out by certified technical consultants and tested by the Trust ICT team and data warehouse team in accordance with Trust ICT procedures and policies.

### Database management

The database will be fully backed up on a daily basis. The back-ups are standardised for all Trust databases within the Trust data warehouse. Backups can be restored at any point by warehouse staff. Data are secondary copies from clinical systems; at any point the participating Trusts can re-extract the data from primary sources. Each site will submit data on a quarterly basis to the database. Data integrity checks will be completed to ensure correct structure is maintained and duplicates are not present.

Once entered on the system, data will not be changed. All access will be ‘read only’ except via exception, approved by the research Informatics Programme Manager and Clinical Leads group.

The database will be managed by the data manager (Ben Glampson) and developer (Abdul Mulla). Any database changes will be controlled by the research informatics Programme manager (Ben Glampson), and sanctioned by the NIHR HIC Cardiovascular scientific steering committee (chaired by Jamil Mayet). All staff are substantive NHS employees at ICHNT.

Data extracts taken for research will be stored within the data warehouse and retained for the period specified in the data request. All information pertinent to the request will be retained and tracked by the data manager.

###

### DATA ACCESS FOR RESEARCH

##### TROP-MALIGNANCY STUDY dataset

All analyses for the TROP-MALIGNANCY STUDY will be completed on fully de-identified data. This includes further de-identification to remove dates.

A designated clinical researcher (Amit Kaura) froze a copy of the database in April 2017, so a static dataset can be used for analysis. This will be retained separately from the live database to allow reproducible analyses.

The study dataset will comprise all patients who had a troponin measured at each of the five academic centres between 2010 (2008 for University College Hospital) and 2017.

Dates will be converted to delta dates, with date zero being the date of the first troponin test. All further dates will be provided as number of days from date zero. Age will be provided in years, at the time of the first troponin test.

Date of death will be converted to the number of days since date zero. All patients will be retrospectively followed up, using routinely collected data on the NHS Spine Application, Summary Care Record, until death or censoring on 1^st^ April 2017.

### Data elements

The database model includes 156 data points, grouped into demographics, emergency department attendance and inpatient episodes, biochemistry, diagnosis, angiography, revascularization, echocardiography and mortality. Diagnostic data will be based on International Statistical Classification of Diseases and Related Health Problems (ICD) discharge codes.
